# Electron-ion coincidence measurements of molecular dynamics with intense X-ray pulses

**DOI:** 10.1038/s41598-020-79818-6

**Published:** 2021-01-12

**Authors:** Xiang Li, Ludger Inhester, Timur Osipov, Rebecca Boll, Ryan Coffee, James Cryan, Ave Gatton, Tais Gorkhover, Gregor Hartman, Markus Ilchen, André Knie, Ming-Fu Lin, Michael P. Minitti, Clemens Weninger, Thomas J. A. Wolf, Sang-Kil Son, Robin Santra, Daniel Rolles, Artem Rudenko, Peter Walter

**Affiliations:** 1 Linac Coherent Light Source, SLAC National Accelerator Laboratory, Menlo Park, CA 94025 USA; 2 J.R. Macdonald Laboratory, Department of Physics, Kansas State University, Manhattan, KAN 66506 USA; 3 Center for Free-Electron Laser Science, DESY, Notkestrasse 85, 22607 Hamburg, Germany; 4 The Hamburg Centre for Ultrafast Imaging, Luruper Chaussee 149, 22761 Hamburg, Germany; 5 European XFEL, Holzkoppel 4, 22869 Schenefeld, Germany; 6 Stanford PULSE Institute, SLAC National Accelerator Laboratory, Menlo Park, CA 94025 USA; 7 Institut für Physik und CINSaT, Universität Kassel, Heinrich-Plett-Strasse 40, 34132 Kassel, Germany; 8 Department of Physics, Universität Hamburg, Jungiusstrasse 9, 20355 Hamburg, Germany

**Keywords:** Atomic and molecular interactions with photons, X-rays

## Abstract

Molecules can sequentially absorb multiple photons when irradiated by an intense X-ray pulse from a free-electron laser. If the time delay between two photoabsorption events can be determined, this enables pump-probe experiments with a single X-ray pulse, where the absorption of the first photon induces electronic and nuclear dynamics that are probed by the absorption of the second photon. Here we show a realization of such a single-pulse X-ray pump-probe scheme on N$_2$ molecules, using the X-ray induced dissociation process as an internal clock that is read out via coincident detection of photoelectrons and fragment ions. By coincidence analysis of the kinetic energies of the ionic fragments and photoelectrons, the transition from a bound molecular dication to two isolated atomic ions is observed through the energy shift of the inner-shell electrons. Via ab-initio simulations, we are able to map characteristic features in the kinetic energy release and photoelectron spectrum to specific delay times between photoabsorptions. In contrast to previous studies where nuclear motions were typically revealed by measuring ion kinetics, our work shows that inner-shell photoelectron energies can also be sensitive probes of nuclear dynamics, which adds one more dimension to the study of light-matter interactions with X-ray pulses.

## Introduction

Electron-ion coincidence measurements, which can provide kinematically complete information associated with each single light-matter interaction event, are a powerful method for studying the response of atoms and molecules to table-top laser pulses and synchrotron radiation^1–4^. They are expected to open up a variety of new possibilities for imaging molecular dynamics at X-ray free-electron lasers (XFELs), in particular, enabling channel-selective and molecular-frame measurements^5^. However, this method has been implemented in only a few experiments at free-electron laser (FEL) facilities to date^6–9^, largely hindered by its requirement of low interaction rates (less than one interaction event per laser shot). With several high-repetition rate X-ray free-electron lasers now coming into operation, electron-ion coincidence measurements are becoming a more feasible approach^10^ that can take full advantage of the envisioned MHz repetition rates in order to significantly increase the information content of the experiments.

In this work, we demonstrate how an electron-ion coincidence measurement combined with a sequential absorption of two X-ray photons from the XFEL pulse and a state-of-the-art theoretical modeling can provide a detailed time-resolved picture of X-ray induced fragmentation of molecules. We observe a shift in the core binding energy of the dissociating N$_{2}^{2+}$ molecular ion, which can be linked to the internuclear separation of the two resulting ionic fragments by measuring their kinetic energies and performing ab initio calculations. Together with a recent demonstration^10^ of photoelectron diffractive imaging of molecular dissociation performed using similar experimental approach but focusing on the changes in photoelectron angular distributions instead of energies, these results demonstrate the general feasibility of coincidence spectroscopy at XFELs and its promising broad perspectives for AMO physics and ultrafast photochemistry.

**Figure 1:**
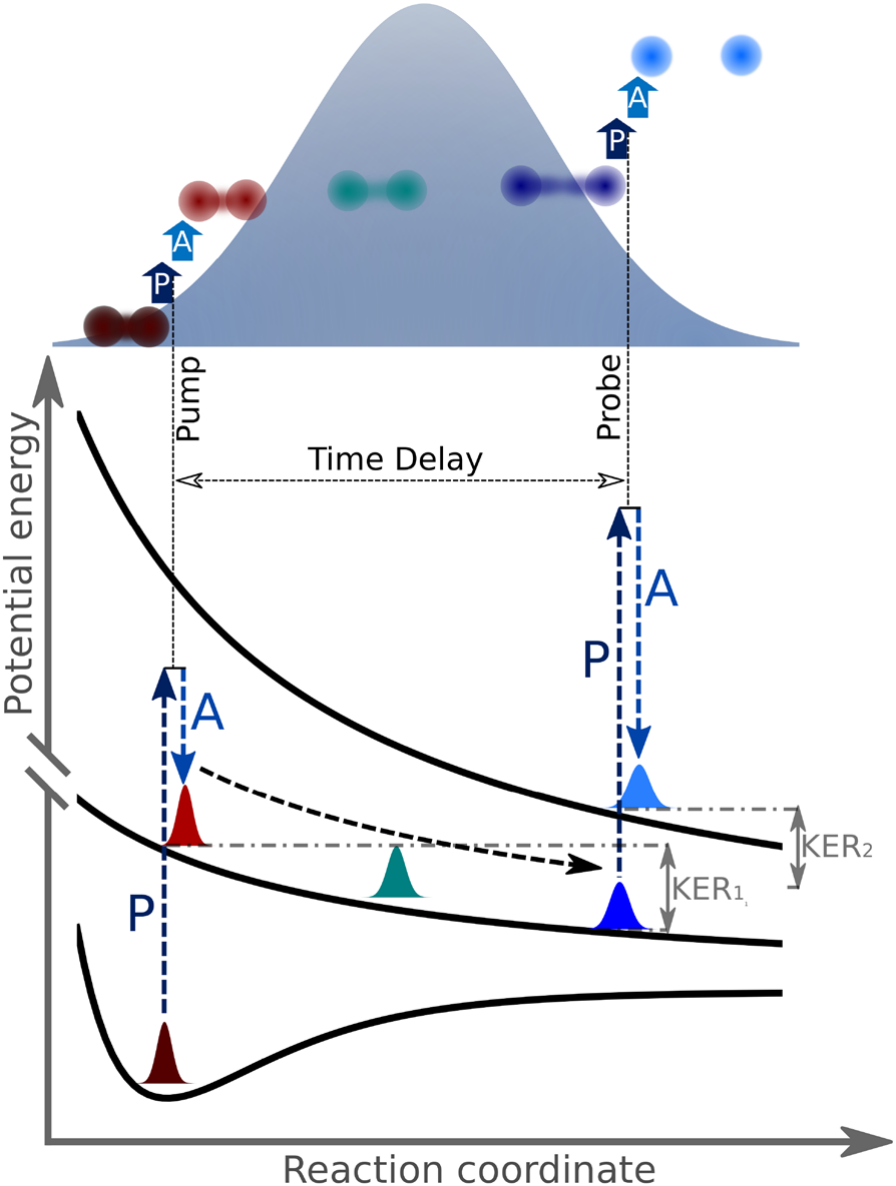
Schematic of the single-pulse pump-probe scheme: Absorption of a first photon early in the FEL pulse leads to the emission of a photoelectron (P) and Auger electron (A) and prepares the molecule in a bound or dissociative dicationic state. In the latter case, the second photoabsorption probes the ensuing dynamics on the dicationic potential energy surface at a variable time delay, encoding the information on the dicationic electronic states in the photoelectron energy, while the information about the time delay is contained in the kinetic energy of the ionic fragments. The final kinetic energy release is the sum of KER$_1$ and KER$_2$.

As shown schematically in Fig. 1, the dynamics in the N$_2$ molecule are first induced and then probed by two sequential photoabsorption events within a *single* FEL pulse. Although conceptually similar to typical pump-probe schemes that employ *two* separate pulses to pump and probe the dynamics, respectively, this circumvents the need to produce two independently controllable X-ray pulses. The figure also shows a sketch of the specific pump and probe processes in the present experiment: a diatomic molecule is core-ionized with the first photoabsorption, which acts as the pump step. After the first photoionization, Auger decay further ionizes the molecule into a dicationic state, which can be bound or dissociative^11–14^. The state of the created molecular ion is then probed by the core-shell absorption of a second X-ray photon. The photoelectron emitted during this second step contains dynamical information about the system prepared by the first step right at the instance of the second photoionization. After the probe step and the ensuing Auger decay, a multiply charged repulsive state is populated, and the molecular ion dissociates into a pair of atomic ions. If the pump step populates a dissociative state and there is a small time delay between pump and probe photoabsorptions, the probe pulse ionizes the dissociating molecular dication during the early stage of its dissociation process when the bond length is short. Because of the large Coulomb repulsion in the multiply charged molecular ion, this short bond length results in a large kinetic energy release (KER) of the final fragments. Conversely, a large time delay between the pump and probe steps will lead to a significantly smaller Coulomb repulsion if the molecular fragments are further apart when their charge is increased to the final charge state in the second photoionization event. This intuitive relation between the observed KER and the molecular bond length, which provides the conceptual basis for the so-called Coulomb explosion imaging approach^15^, has been recently exploited to image the temporal evolution of molecular orbitals by measuring correlated energies of ions and Auger electrons from HCl dissociation in a synchrotron experiment^16^, to time the sequential absorption of several X-ray photons by N$_2$ molecules^17^, and to sort the photoelectron diffraction images in the above-mentioned EuXFEL experiment^10^. In our experiment, the single-pulse pump-probe scheme enabled by the coincidence detection of ions and photoelectrons, along with a quantitative simulation of the relation between the internuclear separation and the observed KER, allows us to probe the evolving core binding energy of the dissociating molecular ion.

Our results obtained with the 120 Hz X-ray pulses at the Atomic, Molecular and Optical (AMO) instrument^18^ of the Linac Coherent Light Source (LCLS) show the great potential of studying light-matter interactions by combining electron-ion coincidence measurement with future MHz XFEL facilities such as the LCLS II where a new permanent end-station will be designated for such type of coincidence experiments. Furthermore, the single-pulse pump-probe scheme can be a relatively simpler alternative to the more complicated two-pulse X-ray-pump X-ray-probe configuration^19,20^, which requires additional machine setup^21^. In contrast to previous studies in which nuclear motions were revealed by fragment ion kinetics^19,20,22^ or Auger electrons^16,23^, the current work demonstrates that core-shell photoelectron energies are also sensitive probes of nuclear dynamics. When combining coincidence detection with the recently available attosecond XFEL pulses^24^, core electrons together with the coincident ions will also be an ideal probe for studying electronic dynamics^25^, as well as their coupling with nuclear degrees of freedom.

## Results

**Figure 2:**
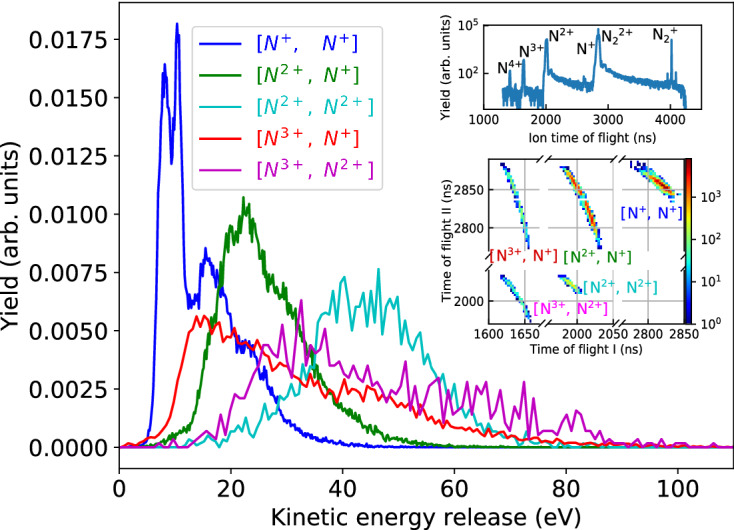
KER for the five strongest ion-ion coincidence channels observed in the interaction of 503-eV, 100-fs (nominal value estimated from the length of electron bunch used for X-ray generation), 10-μJ XFEL pulse with N$_{2}$ molecules. The insets show the corresponding ion time-of-flight spectrum (top) and the photoion-photoion coincidence (PIPICO) map (bottom).

Inner-shell ionization of N$_2$ molecules has been studied extensively^17,26–36^, and many of these studies have made use of various electron-ion coincidence methods, such that the single-photon ionization process is well characterized. However, when subjected to the more intense X-ray pulses from an XFEL, each N$_2$ molecule can absorb more than one photon, resulting in higher charged ions than those observed in synchrotron experiments and more complicated Auger decay pathways^17,26,33,34^. In the present experiment performed at a rather modest pulse energy of $\sim$10 $\mu$J, five ion–ion coincidence channels can be identified, as shown by Fig 2: [N$^{+}$, N$^{+}$], [N$^{2+}$, N$^{+}$], [N$^{2+}$, N$^{2+}$], [N$^{3+}$, N$^{+}$] and [N$^{3+}$, N$^{2+}$]. The former two have also been reported and identified in the synchrotron studies, and their KER spectra are very similar to those observed with synchrotron radiation^28^, suggesting that these pathways mainly stem from single-photon ionization. The other three channels with higher total charge are thus predominantly the product of sequential multi-photon ionization, and their KERs have contributions at significantly higher energy, as expected from the higher Coulomb repulsion in these ionic final states. With our experimental settings, sequential two-photon and single-photon ionizations dominate over higher-order ionizations.

**Figure 3:**
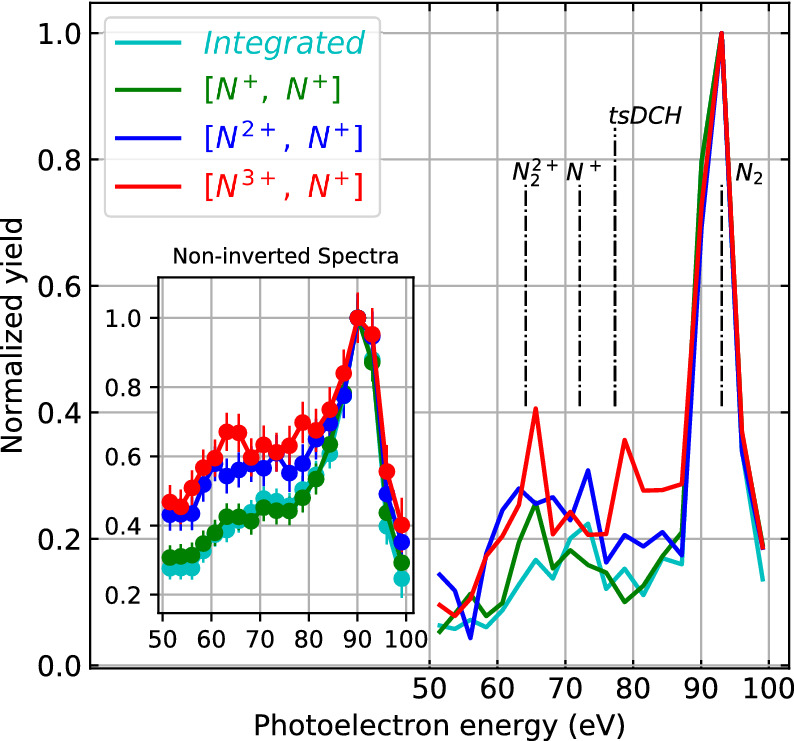
Ion-ion-coincidence-channel-resolved photoelectron kinetic energy spectra. The spectra before inversion (i.e. radial distributions, converted to energy units, of the two-dimensional projections of the momentum distributions) are shown in the inset. Vertical dashed lines mark the calculated photoelectron line positions for various molecular and atomic states of N$_{2}$ (adapted from Ref.^26^). The vertical line labeled N$^+$ corresponds to the transition [$^3$P (2p$^{-1}$) $\rightarrow$ $^4$P (1s$^{-1}$2p$^{-1}$)].

The photoelectron spectra detected in coincidence with three of these fragment channels are plotted in Fig. 3 along with the integrated photoelectron spectrum without any coincidence conditions. The spectra have distinct features, reflecting the physical processes of how the corresponding ionic final states are reached. [N$^{+}$, N$^{+}$] is mainly produced via single-photon absorption, where one electron is ejected by inner-shell ionization and the other by Auger decay. The resulting photoelectron spectrum is dominated by a strong peak at approximately 93 eV, which corresponds to the difference of the photon energy (503 eV) and the N(1s) binding energy in N$_2$ ($\sim$ 410 eV). The broader feature on the left of the major peak, in the range of 60 to 80 eV, is attributed to photoelectrons generated in combination with shake-up processes, where the photoionization step causes additional excitations in the molecule.

For [N$^{2+}$, N$^{+}$], apart from the electron ($\sim$93 eV) ejected by core-shell photoionization, two additional electrons can be ejected either through a double Auger process or Auger decay in combination with one more valence ionization. Both the Auger electrons and the photoelectron from valence ionization have kinetic energies much higher than the energy range shown in Fig. 3. The broad features on the left of the major peak, between 60 and 80 eV, are again attributed to shake-up processes in combination with the first core ionization or to core-shell ionization after valence ionization. In addition, [N$^{2+}$, N$^{+}$] can be created by shake-off processes, where two electrons are emitted by the initial photoionization step. Such processes also contribute to the broad feature on the left of the major peak at 93 eV^31^.

For the [N$^{3+}$, N$^{+}$] fragmentation channel, additional peaks at kinetic energies smaller than 93 eV can clearly be identified. They are attributed to second core-shell photoionizations of the products created by the first photoabsorption step. If the second photoabsorption happens after the Auger decay, ionization of the resulting N$_2^{2+}$ ions contributes to the peak around 64 eV, which will be discussed in details below in connection with the KER of the ion pairs. If the second photoabsorption occurs before the core hole is filled, a double-core-hole state is created, as observed in previous FEL experiments^26,34,37,38^. If the double core holes are located on a single N atom [single-site double-core-hole state (ssDCH)], the kinetic energy of the second photoelectron is about 11 eV^26^. If the double core holes are distributed on two separate N atoms [two-site double-core-hole state (tsDCH)], the second photoelectron contributes to the peak near 79 eV kinetic energy. Given the relatively long pulse duration (100 fs) in the current experiment, the contribution of double-core-hole states is expected to be relatively low. However, as we will show in the photoelectron spectra in coincidence with [N$^{3+}$, N$^{+}$], the contribution from double-core-hole events can be enhanced when selecting only the coincidences with high-KER ion pairs since those are produced predominantly when the two photoabsorption events occurred within a short time interval from each other.

For the more likely scenario where the second photoabsorption occurs *after* the Auger decay, a manifold of dicationic N$_{2}^{2+}$ states can be occupied, some of which are quasi-bound and others are strongly dissociative^11–14^. Depending on when the second photoabsorption occurs relative to the first, it probes different stages in the evolution of those dissociating N$_{2}^{2+}$ molecular ions, and the corresponding photoelectrons ejected in the second step can have kinetic energies ranging from 53 eV to 80 eV, while the ion KER also varies considerably.

**Figure 4:**
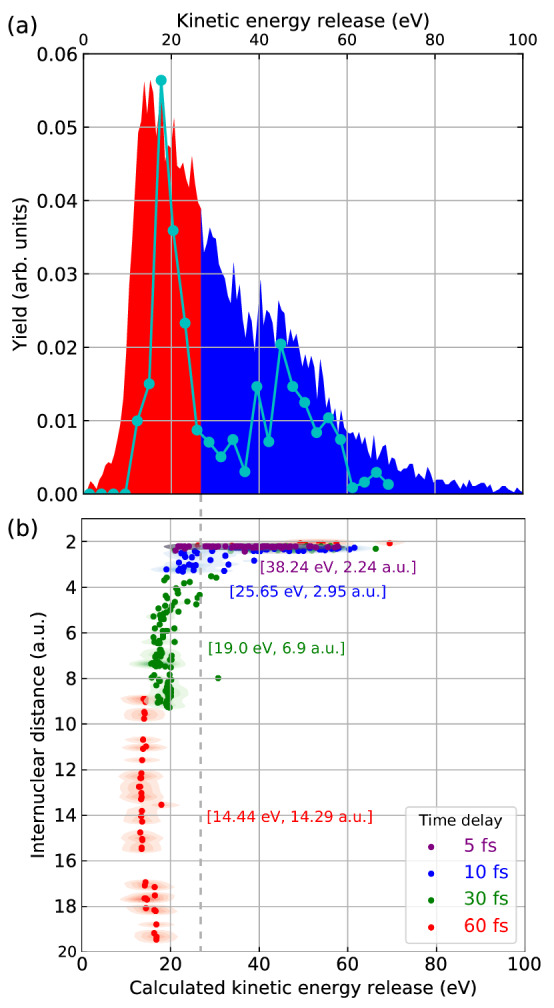
(**a**) Kinetic energy distribution of the [N$^{3+}$, N$^{+}$] coincidence channel. The experimental kinetic energy distribution is divided into 2 regions: a low-KER region (0–27.4 eV, red), and a high-KER region (27.4–100 eV, blue). The calculated KER distribution for an XFEL pulse with Gaussian temporal shape [60 fs pulse duration (FWHM)] is shown by the cyan symbols. (**b**) Calculated KER of the [N$^{3+}$, N$^{+}$] channel for discrete time delays $\Delta t$ between the two photoabsorption steps along with the N$^+$-N$^+$ internuclear distance at the time of the second photoabsorption. The values in square brackets are the average KER and internuclear distance for each time delay (see text). The vertical gray dashed line is the 27.4-eV boundary between the “low-KER” and “high-KER” regions chosen in panel (**a**).

If the second photoabsorption occurs early, the N$_{2}^{2+}$ ion is ionized at a short bond length, resulting in high-KER [N$^{3+}$, N$^{+}$] ion pairs. In contrast, if there is a longer delay between the first and second photoabsorption steps, those molecules in a dissociative N$_{2}^{2+}$ state have evolved to a much larger internuclear distance, resulting in a considerably lower KER. Because of the statistical nature of the two photoabsorption events, the time delay between them can range from zero to the full length of the pulse. Note that the signals from quasi-bound states overlap with those from dissociating N$_2^{2+}$ ions probed by the second photoaborption only at small time delays and not at larger delays, which makes it possible to discuss the state evolution during dissociation. Figure 4a shows the experimental KER distribution for [N$^{3+}$, N$^{+}$] ion pairs, with the low-KER region (KER below 27.4 eV) marked in red, and the high-KER region (KER above 27.4 eV) marked in blue. The cyan data points show the calculated KER of the [N$^{3+}$, N$^{+}$] fragmentation channel assuming a Gaussian temporal pulse shape [60 fs full width at half maximum (FWHM)]. It shows a bimodal structure, with the overall spread in KER in reasonable agreement with the experimental distribution. To further elucidate the structure in the calculated KER distribution, Fig. 4b displays the calculated [N$^{3+}$, N$^{+}$] KER plotted for discrete time delays $\Delta t$ between the two photoabsorption steps along with the N$_{2}^{2+}$ KER at the time of the second photoabsorption. At a time delay of $\Delta t=5\ \mathrm {fs}$, the KER is distributed between 20 eV and 60 eV, and the internuclear separation ranges from 2 a.u. to 2.5 a.u. In other words, at this time delay, N$_{2}^{2+}$ ions have either remained bound or are at the very early stage of their dissociation. For larger time delays, the bound N$_{2}^{2+}$ ion distributions continue to be present in the high-KER and short-bond-length region, whereas the distributions of dissociating N$_{2}^{2+}$ ions are moving to regions of larger internuclear separation (above 2.5 a.u.) and consequently lower KER. The average KER and internuclear separation for each time delay are indicated by the values in square brackets. For time delays larger than 5 fs, data points with bond lengths shorter than 2.5 a.u. are excluded from the calculation of the average values in order to only take into account the dissociating N$_{2}^{2+}$ ions.

Figure  4b directly illustrates that a low KER can be associated with large internuclear distances and corresponding long delay times. According to Fig. 4, [N$^{3+}$, N$^{+}$] ion pairs with a KER in the blue high-KER region are thus produced from photoionization of N$_{2}^{2+}$ ions which are either in a bound state or just started to dissociate, with bond lengths distributed mainly in the range from 2 a.u. to 2.5 a.u. For those dissociating ions, the time delay between the first and second photoionizations is mostly smaller than 30 fs. [N$^{3+}$, N$^{+}$] ion pairs with a KER falling into the red low-KER region are produced from photoionization of dissociating N$_{2}^{2+}$ ions at time delays larger than or equal to 30 fs when the bond lengths are mostly larger than 2.5 a.u.

**Figure 5:**
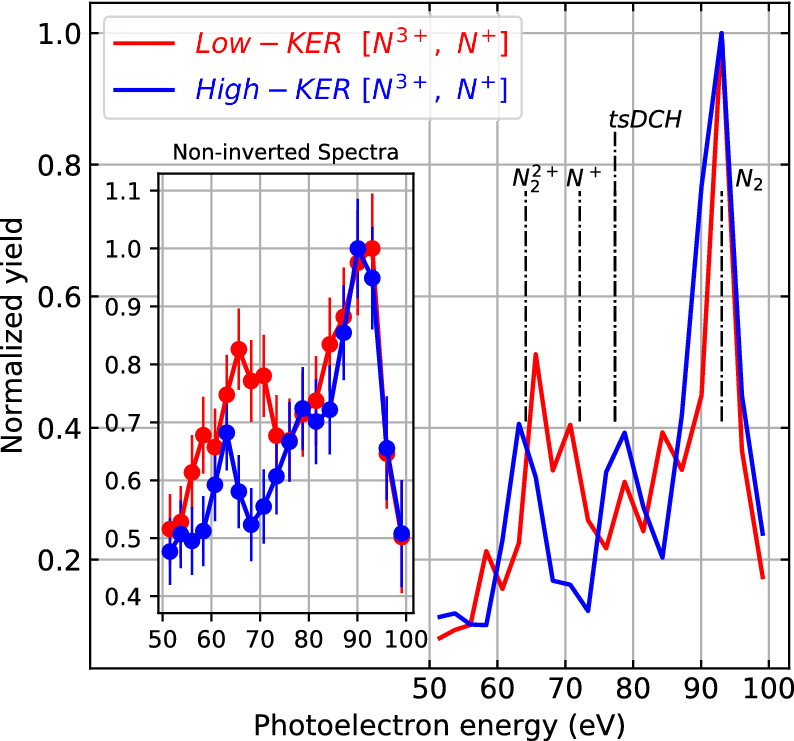
Experimental kinetic energy spectra of photoelectrons detected in coincidence with [N$^{3+}$, N$^{+}$] ion pairs falling into the low-KER and high-KER regions as defined in Fig. 4. The spectra before inversion are shown in the inset. Vertical dashed lines mark the calculated photoelectron line positions for various molecular and atomic states of N$_{2}$ (adapted from Ref.^26^).

The photoelectron spectra detected in coincidence with ion pairs in the low- and high-KER regions respectively are displayed in Fig. 5. As expected, the photoelectron peak from the first photoabsorption appears at 93 eV in both spectra. The peaks at lower kinetic energies, which are attributed to the second photoionization, have different features for the low- and high-KER cases. The peak at 79 eV is enhanced when selecting the high-KER region, which can be contributed by either ionization of N$^+$ (2s$^{-1}$ $\,\rightarrow \,$ 1s$^{-1}$2s$^{-1}$, not shown in the figure) or the second photoelectron in tsDCH production. Since the high-KER ion pairs are produced when the internuclear distance is close to the N$_2$ equlibrium bond length, this enhanced peak cannot stem from the ionization of N$^+$ ions. Instead, this enhanced peak must be attributed to the second photoelectron being emitted in the production of a tsDCH state, which requires short time delays between the two photoabsorptions.

There is a second clear photoelectron peak at approximately 64 eV, whose peak position shows an energy shift depending on the selected ion KER. The maximum of the peak shifts from 63.2 eV to 65.6 eV as the ion KER is reduced. This shift can be attributed to a “dynamic photoelectron line shift”, i.e., a change of core binding energy with bond length. Since the low-KER ion pairs are obtained from ionization of dissociating N$^{+}$-N$^{+}$ pairs at larger internuclear distance, corresponding to large “pump-probe” time delays, the corresponding higher photoelectron energy indicates that N$_{2}^{2+}$ core binding energy becomes smaller as its internuclear distance increases. This lower core electron binding energy is a result of the reduced Coulomb interaction when the neighboring $\mathrm {N^+}$ atom is at larger internuclear distances. In addition, there is a peak near 71 eV, which is present in the low-KER spectrum, but absent in the high-KER one. This peak is from the ionization of N$_{2}^{2+}$ at very large delays when the bond length is so large that the N$_{2}^{2+}$ molecular ion can be approximately regarded as two isolated N$^{+}$ atomic ions.

**Figure 6:**
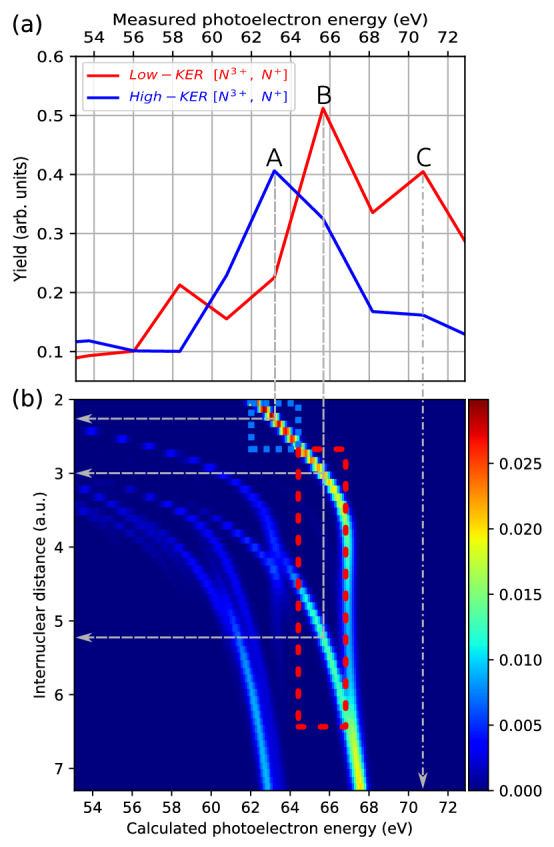
Dynamic photoelectron line shift. (**a**) Experimental photoelectron kinetic energy spectra from Fig. 5, but zoomed into the energy range from 53 eV to 73 eV, corresponding to electrons emitted from N$_{2}^{2+}$ in the second photoabsorption step. (**b**) Calculated photoelectron kinetic energy spectra of N$_{2}^{2+}$ ($1^{1}\Delta _{g}$) as a function of internuclear distance. The color encodes the relative probability for a N$_{2}^{2+}$ photoelectron to have certain energies for a given internuclear distances. The vertical gray dashed lines mark the peak positions (A, B and C) observed in the experimental spectra. The horizontal lines indicate the internuclear distances at which the vertical dashed lines cross the calculated photoelectron lines. The vertical dashed lines at peak C also crosses the calculated photoelectron line, but only at the internuclear distance larger than the range shown in the figure. The calculated photoelectron energy at larger internuclear distances are shown in Fig. 7. The blue and red dashed rectangles mark the regions centered at photoelectron energies of peaks A and B, respectively, with a width corresponding to the experimental energy bin size of 2.5 eV.

**Figure 7:**
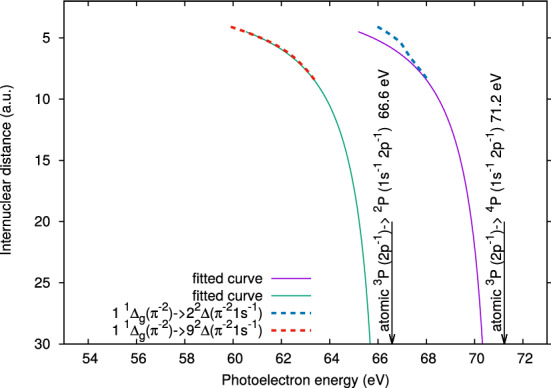
Calculated photoelectron kinetic energy spectra of N$_{2}^{2+}$ ($1^{1}\Delta _{g}$) as a function of internuclear distance. The two dominant absorption lines in Fig. 6 are plotted as dashed lines. The solid curves show the photoelectron kinetic energy at large internuclear distance, which is obtained by fitting Coulomb curves for the large-distance behavior of the involved potential energy surfaces.

N$_2^{2+}$ produced from the first photoionization of N$_2$ and the subsequent Auger decay, can populate a multitude of different states as identified in^39^. Photoelectrons ejected from N$_2^{2+}$ ions at any of these states can contribute to the peaks within the range of 53 to 80 eV in Fig. 5. Together with the broad distribution of intermediate bond lengths, a complete assignment of photoelectron lines is thus impossible. To further investigate the shift of N$_{2}^{2+}$ core electron binding energy at different time delays and internuclear separations, we pick out the $1^{1}\Delta _{g}$ dicationic state, which is one of the dominant Auger channels accounting for $\sim 17$%^39^ of the total Auger yield following the first core ionization. For this state, the calculated photoelectron spectra at different internuclear distances are plotted in Fig. 6b. At equilibrium bond lengths around 2 a.u., there is a single photoelectron line, the energy of which moves to higher values with larger internuclear separations. Above 2.5 a.u., satellite photoelectron lines start to emerge in the lower-energy region due to shake-up processes which, in addition to the ejection of a core-electron, leave the molecular ion in an excited electronic configuration. A more detailed analysis of such shake-up features can be found in Ref.^40^. The photoelectron energy increases with bond length for all of the photoelectron lines, as expected from the reduced Coulomb interaction between the core electron in one N${^+}$ ion and the neighboring N${^+}$ ion. Note that the $1^1\Delta _g$ state considered here asymptotically corresponds to a dissociation into N$^+$ ions in the $^3$P configuration. Beyond an internuclear separation of 6 a.u., the photoelectron spectrum shows two distinct photoelectron lines. When the two N$^+$ ions are so far apart that they can be regarded as two isolated atomic ions, these two photoelectron lines can be associated with the core ionization transition of atomic N$^+$ into two different spin configurations ($^3$P $\,\rightarrow \,$ $^4$P and $^3$P $\,\rightarrow \,$ $^2$P). This can be seen in Fig. 7 which shows the calculated core binding energies for a larger range of internuclear distances, with the two dominant absorption lines in Fig. 6 plotted as dashed lines. To follow the change of binding energies at larger internuclear distances, we have fitted Coulomb curves for the large-distance behavior of the involved potential energy surfaces. The solid lines show the resulting core level binding energies. The arrows indicate the asymptotic values at infinite separation, which can be associated with core level ionization transitions $\mathrm {^3P} \rightarrow \mathrm {^2P}$ and $\mathrm {^3P} \rightarrow \mathrm {^4P}$ in atomic N$^+$.

In order to associate the calculated spectra with the measured ones, the same spectra as in Fig. 5 are shown in Fig. 6a, zoomed into the energy range from 53 to 73 eV. In the following discussion, the $1^{1}\Delta _{g}$ dicationic state can be seen as a model for the “dynamic photoelectron line shift,” since we expect that photoelectron lines shift similarly for most of the populated dicationic states. As illustrated by the vertical gray dashed lines marking the positions of the peaks A and B, photoelectrons ejected from N$_2^{2+}$ ($1^{1}\Delta _{g}$) at small bond lengths can be associated with peak A located at lower photoelectron energy, whereas those ejected from N$_2^{2+}$ ($1^{1}\Delta _{g}$) at larger bond lengths can be associated with peak B located at higher photoelectron energy. The gray line originating from the peak A crosses the photoelectron line at the bond length 2.2 a.u. The blue rectangle, which is horizontally centered at peak A and has the experimental energy bin size of 2.5 eV as its width, covers the bond length range from 2 a.u. to 2.6 a.u. This bond length range agrees reasonably well with the previously determined 2–2.5 a.u. range, which we found to produce high-KER [N$^{3+}$, N$^{+}$] ion pairs. The gray line from peak B crosses the photoelectron line at bond lengths of 3 a.u. and 5.2 a.u. The red rectangle, which is horizontally centered at peak B, covers the bond length range from 2.6 a.u. to 6.3 a.u. This range is consistent with the previous discussion that low-KER [N$^{3+}$, N$^{+}$] ion pairs are produced from N$_2^{2+}$ at time delays larger or equal to 30 fs and bond lengths larger than 2.5 a.u. The vertical line at peak C also crosses the calculated photoelectron line, but only at a bond length beyond the range shown in Fig. 6. According to Fig. 7, this vertical line crosses the calculated line at a bond length and can be assigned to ionization of isolated N$^{+}$ ions [$^4$P (1s$^{-1}$2p$^{-1}$)].

## Discussion

Our experimental and theoretical results reveal the transition from a molecular ion to two isolated atomic ions from the perspective of core electrons. Complementing previous studies where nuclear motions were revealed by measuring ion kinetics or Auger electrons, our experiment demonstrates that the energies of the photoelectrons ejected from the inner shell are also sensitive probes of nuclear dynamics. These results extend the concept of a “molecular frame” measurement, which normally refers to the orientation of one or more molecular axis^3,4,10,41,42^, to the molecular geometry in a more general sense, which is exemplified here by measuring the core-shell photoelectron energy as a function of evolving bond length.

The single-pulse X-ray pump-probe scheme demonstrated in the current work can be a simple alternative to the technically more challenging two-pulse X-ray pump-probe scheme. As illustrated by Fig. 1, it can be used whenever the intermediate state is dissociative and the time delay is thus reflected in the KER of the final ionic fragments, which is often the case for molecules after core ionization. The single-pulse scheme is not limited to two-photon absorption, and can be generalized to the study of other processes involving more than two photons. In that case, the pump photon is still the one that initiates the dissociation, and the probe photon is the one that leads to the time-dependent signals such as photons, photoelectrons or Auger electrons.

In our experimental study, the KER is only divided into two regions, in order to obtain statistically more significant results from the electron-ion coincidence analysis. More KER regions and hence more refined time delays can be chosen for future studies at high-repetition rate XFEL facilities such as the LCLS II and European XFEL. This will allow a more detailed experimental mapping of core electron energy levels, as the one shown by the theory plot Fig. 6b. In the theoretical calculations presented here, only the dicationic state $1^{1}\Delta _{g}$, which accounts for 17% of the population, was picked out to study the dependence of the core binding energy on bond length. Other less populated dicationic states contribute to the photoelectrons in Fig. 5 as well and cannot be disentangled in the current experiment. Given that the dissociative dicationic states show similar trends of core binding energy dependence especially at larger bond lengths, general insight shared by these states can be obtained by the comparison between Fig. 6a,b. A full disentanglement of these dicationic states requires future experiments, where Auger electrons are detected in coincidence with photoelectrons and ions.

## Methods

### Experiment and data analysis

X-ray pulses with a nominal pulse duration of 100 fs and a photon energy of 503 eV were focused by a pair of Kirkpatrick-Baez mirrors into the center of the LAMP^43^ instrument (base pressure below 10$^{-10}$ mbar), where they crossed a pulsed supersonic molecular beam of nitrogen gas. To achieve an interaction rate of less than one ionization event per XFEL pulse, which is necessary to maintain an acceptable level of false coincidences, the X-ray pulse energy was attenuated to $\sim$ 10 $\mu$J at the interaction point.

**Figure 8:**
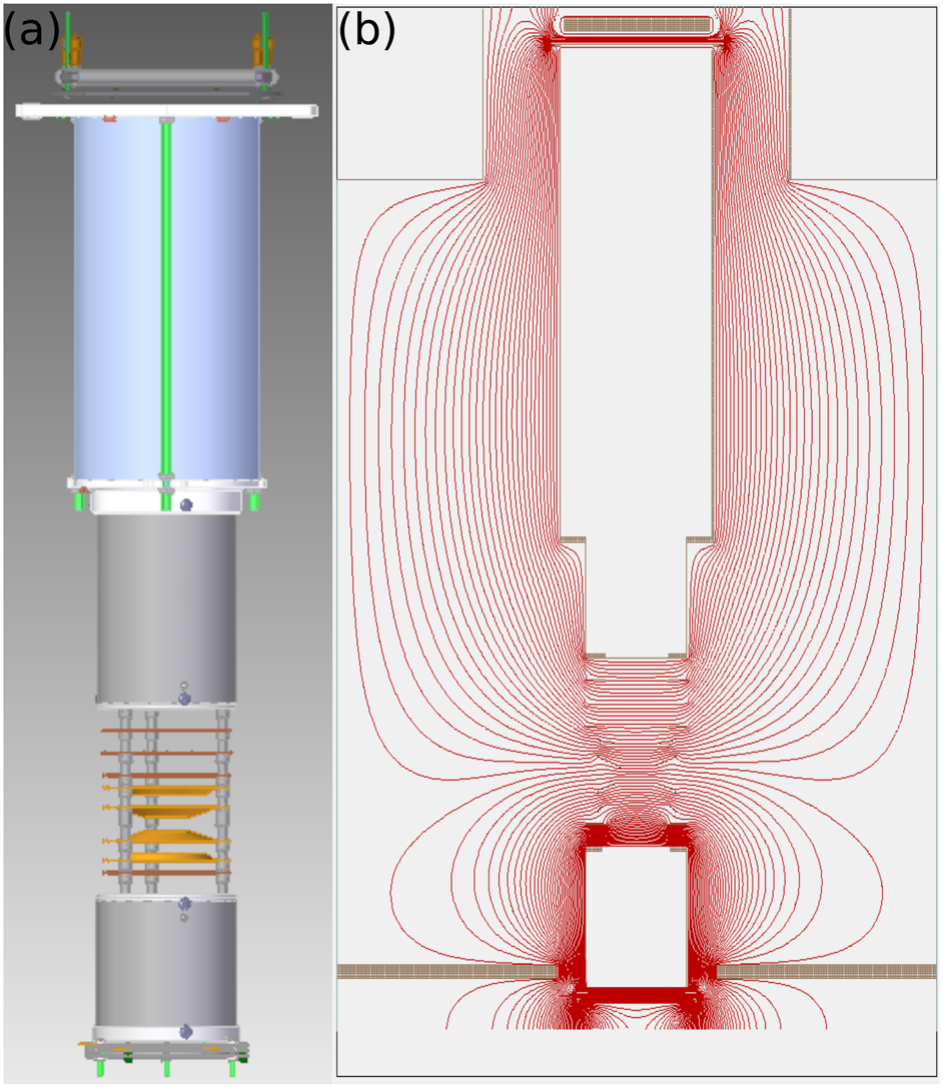
(**a**) 3D CAD rendering of the double-sided electron-ion momentum imaging spectrometer. (**b**) SIMION-simulated equipotential lines of the electric field used in the experiment.

The ions and electrons generated during the interaction between X-rays and N$_2$ molecules were guided onto time and position-sensitive microchannel plate (MCP) detectors by the electric field of a double-sided electron-ion momentum imaging spectrometer^43^, allowing 3-D momentum reconstruction of all interaction products on an event-by-event basis. A CAD rendering and the electric field equipotential lines of the spectrometer are shown in Fig. 8. The ion arm of the spectrometer consisted of an 8 cm long acceleration region and a 42 cm drift region. The electron arm consisted of a 5 cm long acceleration region and a 10 cm drift region. By applying appropriate voltages to the spectrometer electrodes, homogeneous or Velocity-Map-Imaging (VMI) electric fields can be generated for either of the two arms. In the current experiment, the ion arm acceleration region had an almost homogeneous field of approximately 310 V/cm, which results in a 4$\pi$ solid-angle detection efficiency for N$^{+}$ ions with kinetic energies up to 33 eV. The ion detector (with a diameter of 120 mm) is composed of an MCP assembly and a delay-line anode (RoentDek DLD120). The analog detector signals are amplified and digitized. The resulting waveforms are processed using a software constant fraction discriminator. This data is then used to reconstruct the time of flight and hit position on the detector for each particle. From those, the momentum and kinetic energy of each ion are calculated using an empirical formula obtained from SIMION simulations^44^. The ion kinetic energy is calibrated by matching the [N$^{+}$, N$^{+}$] kinetic energy distribution with the one reported in Ref.^28^.

The electron arm of the spectrometer was operated with an inhomogeneous VMI field, capable of detecting electrons with kinetic energies up to 120 eV over 4$\pi$ solid angle. The electron detector (with a diameter of 80 mm) includes an MCP assembly, a phosphor plate, and a high-speed CCD camera (Adimec Opal-1000). For each FEL shot, the camera image is saved together with the ion data for that shot. In the subsequent data analysis, the detector position of each detected electron is found from the camera image by a peak-finding algorithm. The shot-by-shot data is sorted according to the ion(s) recorded in that shot, and corresponding single-shot electron images are integrated to produce a detector image of all electrons detected in coincidence with a certain ion or ion pair. To remove background electrons produced by stray X-rays hitting the spectrometer electrodes, a detector image recorded with the molecular beam turned off, was subtracted from the electron images recorded with the molecular beam on. Note that the electron images recorded with a VMI are a two-dimensional projection of the 3D momentum distributions. The 3D momentum distribution is reconstructed from the VMI image with the “Polar Onion Peeling” inversion algorithm^45,46^. The electron kinetic energy is calibrated by matching the photoelectron peaks with those measured in a previous LCLS experiment^26^. To show the reliability of the reconstruction and the statistics of the experimental data, the photoelectron energy spectra calculated from the reconstructed momentum distributions are shown together with the radial distributions of the VMI image with the radial axis converted to units of kinetic energy. The photoelectrons of interest fall into the kinetic energy range from 50 eV to 100 eV.

The X-ray intensity is about 8.5 $\times$ 10$^{14}$ W/cm$^2$. With the nitrogen photoaborption cross section around 0.4 Mbarn at 503 eV, the probability for single-photon absorption and direct two-photon absorption (estimated according to^47^) is 0.26 and 9.5 $\times$ 10$^{-6}$, respectively. The production of [N$^{3+}$, N$^+$] is mainly achieved by ejecting two 1s electrons followed by Auger decays. The two ejections can be achieved by a sequential two-photon absorption, a combination of single-photon absorption and direct two-photon absorption, or two direct two-photon absorptions, with the corresponding probabilities of the three cases estimated to be 7 $\times$ 10$^{-2}$, 2.5 $\times$ 10$^{-6}$ and 9 $\times$ 10$^{-11}$, respectively. Therefore, to produce [N$^{3+}$, N$^+$] ion pair, sequential two-photon absorption is the dominant process, because it is at least four orders of magnitude more likely to occur than the ones involving direct two-photon absorption.

### Theoretical modelling

Modelling the dissociation dynamics of $\mathrm {N_2}$ upon consecutive core ionizations is challenging due to the many highly excited states that can be populated via Auger decay. To simulate the KER of the fragments, we have therefore chosen an on-the-fly Monte-Carlo approach and conducted molecular dynamics simulations in combination with the XMOLECULE electronic structure toolkit^48,49^. The calculated trajectories started at equilibrium bond length at rest are propagated on the respective potential energy surface. Within a kinetic Monte-Carlo scheme, electronic transitions take place stochastically, according to transition probabilities calculated on the fly. The electronic transition rates for Auger decay are calculated as described in Ref.^49^ on the basis of first-order perturbation theory and employing atomic continuum wave functions.

In contrast to the modelling of X-ray induced ionization and fragmentation dynamics conducted in Ref.^50^, here we have used finite-difference gradients for the forces on the atomic nuclei with configuration interaction (CI) calculations based on Hartree-Fock orbitals employing a 6-31G basis set^51^. The employed configurational space includes all spin- and symmetry-adapted configurations distributing valence electrons within the valence-orbital space ($2\sigma _g 2\sigma _u 3\sigma _g \pi _u \pi _g 3\sigma _u$), and we restrict the calculation to the 100 lowest electronic states per irreducible representations, taking into account only singlet or doublet states. The calculations are conducted in $C_{2v}$ symmetry and, for core hole states, we employ orbitals obtained in an SCF calculation with localized core holes.

The rate for a specific CI state is obtained by only taking into account the dominant configuration of the CI expansion. When we conduct an ioniziation step to another electronic configuration, we randomly pick a new CI state from the manifold of ionized CI states with probabilities chosen according to the population of the CI state in the respective configuration. During the propagation of the molecular geometry, we monitor the character of the electronic state by calculating wave-function overlaps and hop to another CI state at so called “trivial” crossings of the potential energy curves^52^.

We have performed simulations for fixed delay times (5, 10, 15, 20, 25, 30, and 60 fs) between two photoionizations. The KER histogram for a finite pulse duration is then obtained by integrating over delay times as described in the following. The probability to observe a specific KER due to sequential two-photon ionization during an X-ray pulse is given by

1 $$\begin{aligned} P(E_{\text {KER}}) = \int _0^\infty d \Delta t \, \frac{dP^{(2)}(\Delta t)}{d \Delta t}\, P(E_{\text {KER}}| \Delta t), \end{aligned}$$

where $P(E_{\text {KER}}| \Delta t)$ is the conditional probability distribution for the KER given a particular time $\Delta t$ between the two photoabsorptions and $dP^{(2)}(\Delta t)/d\Delta t$ is the probability of having a delay time $\Delta t$. We have performed simulations of the fragmentation with fixed delay times between the ionization steps. These simulations reveal the probability distribution of kinetic energies for a fixed delay time $P(E_{\text {KER}}| \Delta t)$. Employing a Gaussian temporal pulse shape with a photon flux $f(t)=F/(\sqrt{2\pi }\tau ) \exp (-t^2/(2 \tau ^2))$, with a fluence *F*, a width of $\tau$ (standard deviation), and assuming a constant cross section $\sigma$, the timing probability $dP^{(2)}(\Delta t)/d \Delta t$ can be calculated as

2 $$\begin{aligned} \frac{dP^{(2)}(\Delta t)}{d \Delta t }= & {} \sigma ^2 e^{-\sigma F} \int _{-\infty }^\infty d t_1 \, \int _{t_1 + \Delta t}^\infty d t_2 f(t_1) f(t_2) \nonumber \\= & {} \frac{F^2 \sigma ^2 e^{-\sigma F}}{2\sqrt{\pi \tau }} e^{-\Delta t^2/(4 \tau ^2)} . \end{aligned}$$

To obtain KERs for a Gaussian pulse, we have interpolated the calculated kinetic energy distributions $P(E_{\text {KER}}| \Delta t)$ along $\Delta t$ and numerically evaluated the integral in Eq. (1) using the analytical formula in Eq. (2). A parameter relevant for this evaluation is the X-ray pulse duration. It should be stressed that the 100 fs value quoted in the experiment description above is the “nominal” value for the pulse duration, estimated from the length of the electron bunch used for X-ray generation. Previous studies [(e.g., Ref.^53^)] at the Linac Coheret Light Source revealed that the real measured pulse duration of the X-ray pulse was shorter, typically on a level of about 2/3 of this nominal value. We have chosen the 60-fs pulse duration for the simulation, which is close to the expected experimental value and was found to reproduce the experimental kinetic energy release reasonably well, as shown in Fig. 4a.

To calculate photoelectron spectra as a function of internuclear distance, we follow the procedure described in Ref.^40^. In particular, we employ CI expansions for initial dicationic and final tricationic, core-ionized states and calculate the ionization cross sections into all tricationic channels employing an atomic continuum wave function. For the calculation of photoelectron spectra, we employ the same configurational space as specified above in combination with a larger basis set [6-311G(d,p)]^54^.

## Data Availability

The data that support the findings of this study are available from the corresponding authors upon reasonable request.
